# A Study on Ammonium Chloride Dendrite Tip Kinetics: The Importance of the Solid–Liquid Density Change and Interfacial Kinetics

**DOI:** 10.3390/ma17112768

**Published:** 2024-06-06

**Authors:** Nashmi Alrasheedi, Mihaela Stefan-Kharicha, Ibrahim Sari, Mahmoud Ahmadein, Abdellah Kharicha

**Affiliations:** 1 Mechanical Engineering Department, Imam Mohammad Ibn Saud Islamic University, Riyadh 11564, Saudi Arabia; 2 Metallurgy Department, Montanuniversitaet of Leoben, Franz-Josef-Str. 18, A-8700 Leoben, Austria; 3 Department of Production Engineering and Mechanical Design, Tanta University, Tanta 31512, Egypt

**Keywords:** dendrite growth, tip kinetics, ammonium chloride, NH_4_Cl, solutal convection, Ivantsov’s solution

## Abstract

Ammonium chloride (NH_4_Cl) has been extensively studied as a transparent analogue for investigating the solidification of metals due to its distinctive properties and the simplicity of the experimentation. Furthermore, NH_4_Cl exhibits a striking resemblance in solidification behavior to the majority of binary eutectic alloy systems, rendering it a valuable model for studying phase transition phenomena. Experiments conducted on ammonium chloride are frequently employed to validate numerical models for predicting grain structures, macrosegregation, and the columnar-to-equiaxed transition (CET). This latter phenomenon arises due to differences in the velocities of columnar dendrite tips and the liquidus isosurface. However, the kinetics of dendrite tip growth, as a function of supersaturation, remains poorly understood for this commonly used alloy. The objective of this study was to utilize the available experimental data in conjunction with Ivantsov correlations to shed light on the ambiguous kinetics. The results indicate that when considering the crystal–melt density ratio, the Ivantsov solution offers a good correlation. Furthermore, incorporating a moderate interfacial kinetic coefficient enhances the correlations further. This correlation can be implemented in numerical models, which will aid in the determination of the columnar front, the columnar-to-equiaxed transition, and the equiaxed growth velocities.

## 1. Introduction

The solidification of metal alloys incorporates several phenomena interacting in a very complex manner. For instance, crystal formation and growth, grain transport, liquid convection, phase transformations, etc., affect the properties and the formed structural defects in the final products. Experimental investigations of micro/macro-phenomena during the melting and solidification of metallic systems are very hard and exhaustive, since these systems are opaque and their melting points are relatively high. Alternatively, metallurgists and materials researchers conducted necessary investigations on the low melting temperature transparent systems. Among them, the ammonium chloride (NH_4_Cl) was the most common one. It is relatively inexpensive, non-toxic, and readily available, making it a convenient model system for laboratory experiments. Its transparent nature and well-defined phase behavior simplify experimental setup and data analysis, allowing researchers to focus on studying fundamental solidification phenomena.

NH_4_Cl solidifications have allowed researchers to directly observe the solidification process through optical techniques [1,2,3] or particle image velocimetry (PIV) [4,5,6]. This transparency facilitates the real-time visualization of phase transitions, dendritic growth, and microstructural evolution during solidification, providing valuable insights into the underlying mechanisms. By controlling experimental conditions, such as the temperature gradient and cooling rate, researchers can induce dendritic growth in NH_4_Cl crystals that closely resembles the dendritic growth of metallic alloys. Moreover, NH_4_Cl solidification is accompanied by convective flow similar to that observed in the case of metal solidification.

Furthermore, NH_4_Cl provides precise benchmarks for validating theoretical predictions and computational simulations of solidification kinetics and microstructural evolutions [1,2,3,4,5,6,7,8,9,10,11,12,13,14,15,16,17,18,19,20,21,22,23,24,25,26]. Numerical simulations, based on principles of the heat transfer, mass transport, and phase equilibria, are commonly used to model solidification processes in NH_4_Cl and other materials. By comparing experimental data from NH_4_Cl solidification experiments to simulated results, researchers can validate the accuracy and predictive capabilities of numerical models, identifying areas for improvement and refinement.

Once initiated, the growth of NH_4_Cl dendrites proceeds through the attachment of NH_4_^+^ and Cl^−^ ions at the dendrite tips, followed by the propagation of dendritic arms as the solidification continues. The branching morphology of NH_4_Cl dendrites arises from the anisotropic growth kinetics, where certain crystallographic directions exhibit faster growth rates than others. This results in the formation of dendritic structures with elongated branches extending outward from the nucleation sites.

Kurz [27] suggested an approximation for calculating the tip Peclet number (Pe) by inversing the Ivantsov solution. However, Sari et al. [28] reported that the accuracy of the Kurz approximation for estimating Pe, with respect to the Ivantsov function, showed a relatively high error of approximately 20% when estimating Pe at low supersaturation values, which is significant and cannot be negligible. Therefore, Sari et al. [28] proposed a refined approximation specifically tailored to compute the Peclet number for a given supersaturation, building upon the Kurz approach. This new approximation was in good agreement with the Ivantsov function for a wide range of supersaturation values. In addition, Sari et al. [29] proposed an innovative model for precisely predicting secondary dendrite arm spacing (SDAS) based on the tip velocity and cooling rate during the directional solidification of various Pb-Sn alloys. This model conceptualized a growing cylinder within a liquid cylindrical envelope. The initial radius of the cylinder was assumed to match the dendrite tip radius, while the cylindrical envelope maintained a constant radius proportional to the dendrite tip diffusion length. Their findings indicated that lower initial concentrations and slower cooling rates resulted in the formation of coarser arms. Conversely, the predicted SDAS values decreased with higher initial concentrations. The model’s validation showed an excellent correlation with available measurements for SDAS and tip velocity predictions.

McFadden and Coriell [30] extended the Ivantsov solution for free dendritic growth in an undercooled melt to include the influence of density change upon solidification, focusing on an axisymmetric paraboloidal dendrite. Emmerich [31] similarly expanded this solution for a two-dimensional parabolic plate dendrite. In this extended framework, the growth Peclet number, Pe, governing the tip growth, not only becomes dependent on the dimensionless melt undercooling, but also on the relative density change, β. The study revealed that the magnitude of melt flow induced by density change is approximately of the order of β, with the flow velocity diminishing as the distance from the dendrite increases. Despite the significant role of density-induced flow, the extended Ivantsov solution does not incorporate the Prandtl number or, more specifically, the liquid viscosity.

Sun et al. [32] investigated the impact of density variation between the solid and liquid phases on the free dendritic growth of a pure material within an undercooled melt through phase-field simulations. The dendritic growth is modeled as two-dimensional inside of a Hele-Shaw cell. Their findings were in good agreement with Emmerich’s analytical solution [31], particularly for the dependence of the dendrite tip growth Peclet number on the relative density change. Qin et al. [33] utilized synchrotron X-ray radiography and tomography to investigate the growth dynamics of primary Al_3_Ni intermetallic phases in an Al-15%Ni alloy under the pulse electromagnetic fields. Their study, supported by multiphysics modeling, revealed that increased peak magnetic flux density enhances forced convection at the liquid–solid interface, significantly affecting the growth velocity and orientation of the Al_3_Ni phase. This results in the development of full dendritic structures at higher magnetic flux densities, specifically at a peak flux of 1.5 T. This comprehensive approach provides a robust theoretical framework for understanding intermetallic phase growth dynamics during solidification under pulse magnetic fields.

The columnar-to-equiaxed transition (CET) is one of the most investigated phenomena. It is a critical phenomenon observed during the solidification of metallic alloys. To predict the CET, the columnar primary dendrite tip front must be explicitly tracked. The CET occurs only if necessary undercooling for the nucleation of equiaxed grains can be achieved ahead of the columnar tips, i.e., when the advancement of the columnar tips is slower than the thermal isotherm. In other words, predicting CET in the solidification of NH_4_Cl-H_2_O alloys can only be achieved if the dendrite tip kinetics is accurately known. However, the kinetics of dendrite tip growth as a function of supersaturation remains poorly understood. The objective of this study is to utilize the available experimental data in conjunction with Ivantsov correlations to shed light on the ambiguous kinetics. A correlation for the NH_4_Cl dendrite tip kinetics was suggested and the underlying physics was explored. Different models have also been numerically investigated using the software MATLAB R2022b.

## 2. Description of the Experimental Data

Chan et al. [34] examined the “slow” and “fast” dendritic development of NH_4_Cl crystals in aqueous solution at different supersaturation levels. Their goal was to accurately determine the range of occurrence of the various stationary and nonstationary dendritic growth forms. It was shown that the crystal’s surface is deformed in specific ways due to anisotropic interfacial free energy and an anisotropic rate constant, and that the various dendritic forms are the outcome of the protrusions of different modes of deformation being enhanced by the diffusion field.

There are three primary super saturation value ranges where various stationary dendritic growth types were observed. Only crystals of the dendritic structure, in which the side arms and main stem develop in the <100> directions, were found in the lowest range. However, the dendritic forms <110> and <111> were detected in the intermediate and upper supersaturation ranges, respectively. It was also found that the <111> form has its main stem in the <111> direction, whereas the <110> form has its side arms in the <100> directions and its main stem in the <110> direction.

**Figure 1:**
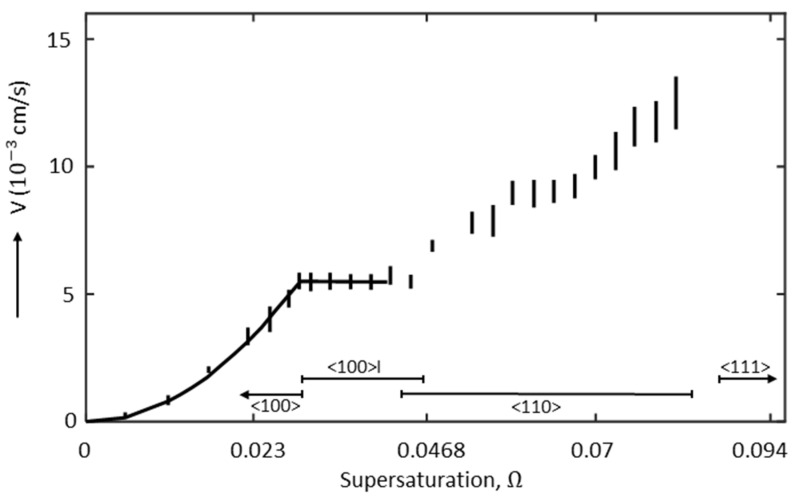
Growth rate of the dendrite tip as a function of supersaturation at T = 25 °C, more precisely showing the extent of occurrence of the stationary <100> and <110> forms and the non-stationary <100>I form with periodic splitting of the tip. Reproduced with permission from [34].

Measurements were made in carefully controlled settings to calculate the growth rate in these ranges as a function of supersaturation. The behavior at T = 25 °C is depicted in Figure 1. Along the abscissa, different growth forms’ zones of existence were also indicated.

The <100> dendritic form is represented by the initial portion of the curve, which starts from the left. A parabola can be used to fit the growth rate V at low supersaturation (Ω<0.03):

(1) $$V=0.06\cdot \Omega^{2}\;\mathrm{with}\;\Omega =\frac{C_{eq}(T)-C_{0}}{C_{eq}(T)-C_{s}},$$

where $C_{eq}$ is the equilibrium concentration, and $C_{0}$ and $C_{s}$ are the initial and solidus concentrations, respectively.

The growth rate curve exhibits a kink during the change from the stationary dendritic form to the non-stationary form, with periodic tip splitting and dependent tip splitting. Then, in the zone with periodic splitting (Ω~0.03–0.0468), the growth rate shows no sign of dependency on supersaturation. At higher supersaturation (Ω>0.0468), the <110> dendritic form appears and the growth rate increases again. However, it is unclear if this second transition occurs with a kink or a tiny discontinuity in the growth rate curve. Figure 1 shows that the range of the <111> form cannot be defined by simple growth laws, like linear or parabolic laws. The middle of the range has a definite and repeatable flap plateau. The change from the <110> to the <111> dendritic form happens at even higher supersaturation (Ω>0.086), accompanied by a significant discontinuous surge in growth.

When converting the supersaturation in undercooling, the transition between <110> and <111> occurs at undercooling in the order of 25 °C, which is not believed to be reached in macroscale experiments, such as those used for the validation of numerical models [1,2,3,4,5,6,7,8,9,10,11,12,13,14,15,16,17,18,19,20,21,22,23,24,25,26].

Therefore, the current investigations are limited to the parabolic range corresponding to the <100> dendritic structures before the occurrence of the <100>I form with the periodic splitting of the tip (Figure 1).

## 3. The Ivantsov Solution for the Growth of NH_4_Cl

One of the foundational contributions to dendritic growth modeling comes from Ivantsov, who provided an elementary mathematical treatment to the steady-state transport process at the solid–liquid interface via diffusion. His work laid the groundwork for understanding the dendritic growth under diffusion-limited conditions [35,36]. In the basic model proposed by Ivantsov, it is assumed that no flow exists in the liquid, and a boundary condition exists at an infinite distance from the solid–liquid interface, where the field parameter (such as concentration or temperature) is at some nominal value. 

**Figure 2:**
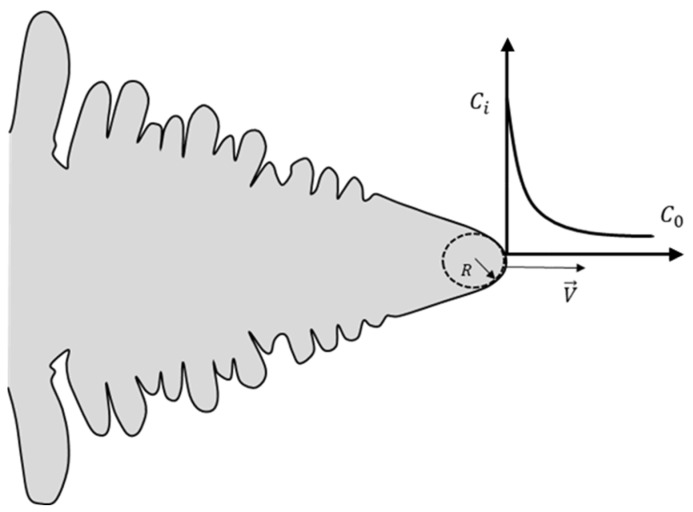
Schematic of solute concentration ahead of a paraboloid of revolution growing at the speed V.

These models are particularly applicable to isolated dendrites with no interfering neighbors (Figure 2). For thermal dendrites growing in a pure substance, the transport of heat at the solid–liquid interface determines the growth rate. Hence, Ivantsov’s diffusion transport model has been applied to the temperature field to determine the growth conditions. For solutal dendrites (found in metallic alloys), the transport of both solute and heat through the solid–liquid interface governs the growth rate. Lipton, Glicksman, and Kurz [37] demonstrated how the Ivantsov mathematical model could be deployed to treat both the heat transport and solute transport problems for unconstrained equiaxed grains. However, Trivedi and Kurz [38] showed that if the temperature field is known or assumed a priori (for example, in the directional solidification scenario), then only the Ivantsov solution is needed to treat the solute transport problem. This can exactly be applied to the present study where individual dendrites grow in a uniform temperature field.

The Ivanstov equation gives the value of the tip Peclet number (Pe) from the supersaturation Ω:

(2) $$\Omega =Pe\;e^{Pe}E_{1}(Pe),$$

with Ω given in Equation (1) and $Pe=\frac{RV}{2D}$. Here, R is the dendrite tip radius, D is the solute diffusivity, and E_1_ represents the exponential integral function:

(3) $$E_{1}\left(x\right)=-\int_{-x}^{+\infty }\frac{e^{-t}}{t}dt.$$

An additional selection criterion is necessary to determine the operating conditions, specifically the combination of radius and growth rate at the dendrite tip. It assumes that the tip selection parameter remains constant for all the materials under all the conditions. For ammonium chloride dendrites, it was experimentally determined [39,40] that the product of R and V is relatively constant:

(4) $$R^{2}V=\beta ,\;\mathrm{with}\;\beta =12\pm 2\;\mu m^{3}s^{-1},$$

where β is the relative density. This relation can be modified to extract a unique dendrite tip radius and growth velocity from the previously determined Peclet number:

(5) $$R=\frac{\beta }{2DPe}\;\mathrm{and}\;V=\frac{4D^{2}{Pe}^{2}}{\beta }$$

**Figure 3:**
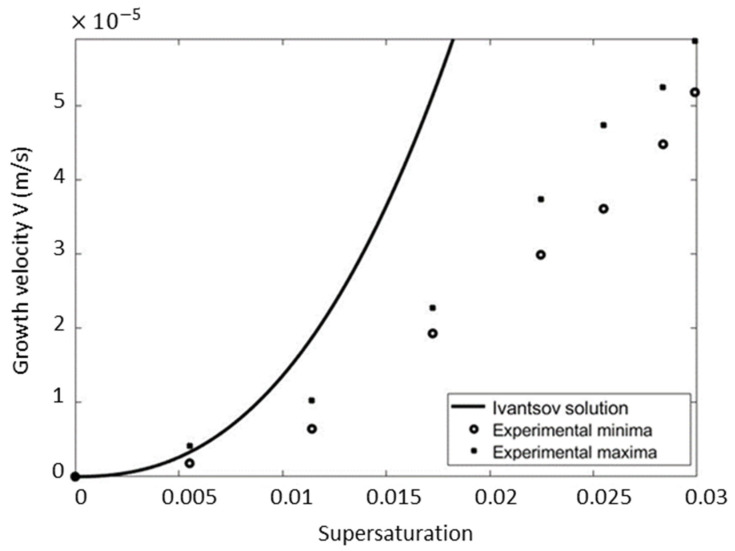
Disagreement between the measurements [34] and Ivantsov solution of the dendrite tip growth rate.

Using data reported in Table 1, the predicted tip velocity is plotted in Figure 3 versus the minimum and the maximum reported experimental data.

It can be shown that the Ivantsov solution approaches the experimental data only for Ω<0.0065. For the higher values, the Ivantsov’s solution deviates strongly from the measurements. The mean percentage error is far larger than 100% (see Table 2). 

## 4. Impact of the Density Change

The solidification of an undercooled melt involves a local change in density, typically with the solid being denser than the liquid. This change, which is usually a few percent for simple metals, induces a flow in the liquid towards or away from the solid–liquid interface, depending on the sign of the density variation. In the case of ammonium chloride, the density ratio depends on both temperature and concentration. For example, at 25 °C, depending on the concentration, the liquid density varies from 1060 to 1090 kg/m^3^. With the solid density being 1520 kg/m^3^, the density ratio is in the following order:

(6) $$\frac{\rho_{s}}{\rho_{l}}\approx 1.4,$$

where $\rho_{l}$ and $\rho_{s}$ are the densities in the liquid and solid, respectively. This mass advection carries heat along with it, even in the absence of the thermally induced natural convection in the liquid phase. Consequently, the aspect of the growth process that relies solely on pure diffusion becomes inadequate and must be coupled with an accurate description of hydrodynamic phenomena. This became apparent in the experiments conducted by Glicksman et al. [42] aboard the space shuttle. In a microgravity environment, where the natural convection was suppressed, they studied the growth of a free dendrite. Similarly, but not as pronounced as in Figure 3, experimental data indeed exhibited deviations from the predictions of the Ivantsov diffusional theory [42]. 

McFadden and Corriel [30] have considered the effects of fluid flow, due to volume contraction or expansion, after being solidified in the Ivantsov analysis of an isolated isothermal developing into a supersaturated liquid. They extracted an analytical solution of the Navier–Stokes equation for an axisymmetric paraboloid dendrite. The flow intensity was found to be directly proportional to the relative density change $\beta =\frac{\rho_{s}}{\rho_{l}}-1$, and it disappears far from the dendrite surface. The Ivantsov expression in the absence of fluid flow, modified for the nonzero β, takes the following form:

(7) $$\Omega =\left(1+\beta \right){Pe}^{1+\beta Pe}e^{Pe}\Gamma \left(-\beta Pe,Pe\right),$$

where Γ is complementary incomplete gamma function:

(8) $$\Gamma (a,x)=\int_{x}^{\infty }e^{-t^{a-1}}dt,$$

where a is the lower limit of the regularized complementary incomplete gamma integral. Once the Peclet number is deduced from Equation (7) for a specific supersaturating, the growth velocity of the dendrite tip can be extracted using the same procedure as that shown in Equation (5).

**Figure 4:**
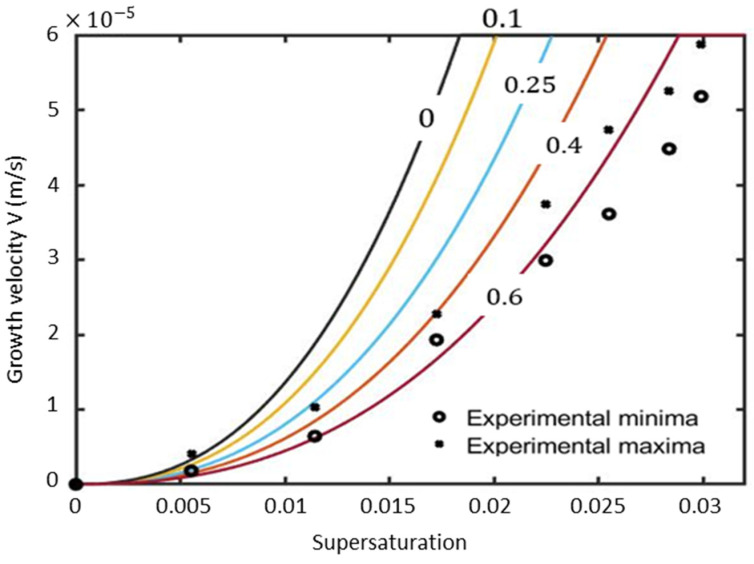
Comparison between experiments [34] and the predicted tip growth velocity using Equation (7) for various relative density changes for β = 0, 0.1, 0.25, 0.4, and 0.6 presented with black, yellow, blue, orange, and garnet solid line colors, respectively.

The growth velocity for different values of relative density change are plotted in Figure 4. The predicted velocity is in good agreement for supersaturations smaller than 0.011 only, and remarkably only for relative density changes close to the solidification of NH_4_Cl (β=0.4). The mean percentage error for supersaturation is only 14% smaller than 0.0125 and 42% for the entire data set (Table 2).

For small β < 0.1, the solution is very close to the classical Ivantsov solution. This was analytically noticed by Mac Fadden and Corriel, [30], through plotting the dimensionless undercooling versus Peclet number (Pe) for different β values. The results show that the effect of varying β in the range of −0.1 to 0.1 was found to be very small. During the solidification of metals, β values are in the order of 0.05; thus, the effects of density change are insignificant. 

The fact that predictions with β=0.4 departs strongly from the experiments for larger growth velocities (Figure 4) is a strong indication that interfacial kinetics might be responsible for this disagreement.

## 5. Importance of Interfacial Kinetics

Raz et al. [43] and Tanaka et al. [44] have demonstrated that the concentration field surrounding a growing NH_4_Cl dendrite can be mapped using the interference microscopy approach. They showed that the fluid in contact with the growing surface of the dendrite has a finite supersaturation, and that this supersaturation is very nonlinearly related to the growth rate. They observed the concentration field around a dendrite growing from a supersaturated NH_4_Cl aqueous solution. They found that the difference between the concentration at the tip and that at the equilibrium increased linearly with the tip velocity, implying that the kinetic term KV dominates the interfacial behavior of the growing dendrite. 

Assuming that the equilibrium hypothesis is violated at the interface, we obtain the following:

(9) $$C_{i}=\left(1+\beta \right)KV+C_{eq},$$

where K is the kinetic coefficient. 

If one assumes the following:

(10) $$KV\ll C_{s}-C_{eq},$$

the kinetic supersaturation $\Omega_{K}$ can be related to the supersaturation Ω, as shown below:

(11) $$\Omega_{K}=\frac{C_{i}(T)-C_{0}}{C_{i}(T)-C_{s}}\approx \Omega -\left(1+\beta \right)\frac{C_{S}-C_{0}}{{\left(C_{eq}-C_{s}\right)}^{2}}KV$$

Equation (9) can simply be modified as the following:

(12) $$\Omega =\left(1+\beta \right){(Pe}^{1+\beta Pe}e^{Pe}\Gamma \left(-\beta Pe,Pe\right)+\frac{C_{S}-C_{eq}}{{\left(C_{eq}\left(T\right)-C_{s}\right)}^{2}}KV)$$

**Figure 5:**
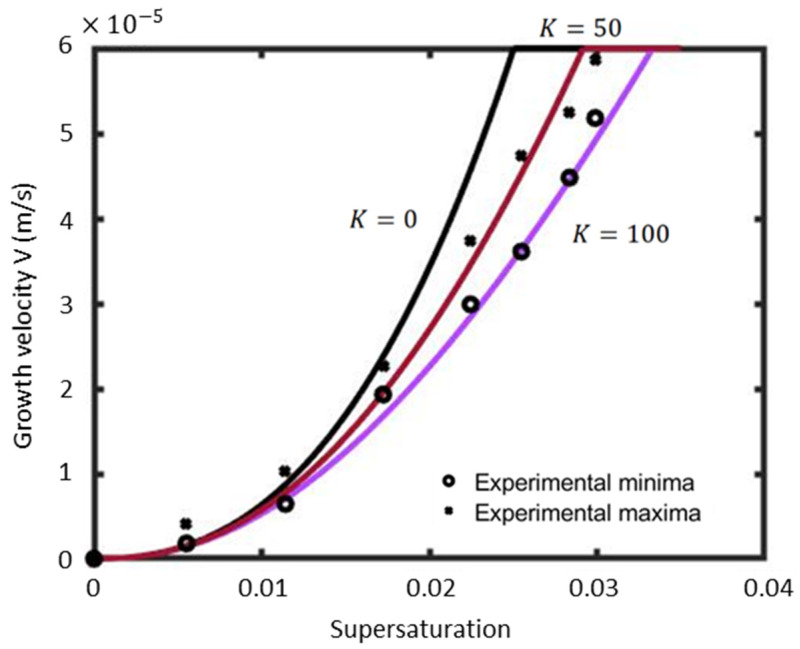
Comparison between tip velocities from experiments [34] and from predictions for a constant β = 0.43 and various interfacial kinetic coefficients K.

Using Equation (10), the growth velocity of the dendrite tip is plotted for different kinetic coefficients, as shown in Figure 5.

It is obvious that the experimental minimum seems to be fitted well with the predictions for a value of K = 100 on the entire investigated supersaturation range with only a 2% error (Table 2). Nonetheless, the experimental data of Tanaka and Sano [43] suggested a value of K = 175, which is very close to the present value. It was found that $KV\approx 6\times 10^{-4}$ and $C_{s}-C_{eq}\approx 0.72$, which fulfills the assumption of Equation (9). It is also noteworthy that K has almost no impact on the resulting velocity at low values of supersaturation (Ω<0.0075). However, its effect is more pronounced at larger values of the supersaturation.

Equation (12) can thus consistently be applied to predict dendrite tip growth velocity of the hypo- and hyper-eutectic alloys of the equilibrium phase diagram of NH_4_Cl-H_2_O.

## 6. Conclusions

The growth kinetics of NH_4_Cl was described for small Peclet numbers corresponding to undercooling smaller than 9 K or supersaturation smaller than 0.032. The classical Ivantsov solution provided a good correlation only for supersaturation smaller than 0.0065. By taking into account the density change between the solid and liquid, the modified equation proposed by McFadden and Coriell [30] was able to fit the data for supersaturation up to 0.017. This modified equation was further amended to account for the existence of interfacial kinetics discovered by Tanaka [43]. The experimental data range was best fitted using a kinetic coefficient ranging from 50 to 100, which is relatively close to the one proposed by Tanaka et al. [43]. The fact that an excellent agreement was found using the physical density ratio (1.43) and an experimentally determined kinetic coefficient promotes the validity of the currently proposed correlation.. The suggested correlations are able to determine at which undercooling/supersaturation the dendrite tip will be located. Solidification and solute redistribution occur in a specific location only when the dendrite tip reached this region. The verified correlation can be implemented within the volume-averaged models to accurately calculate the dendrite tip velocity, which represents a very critical variable in predicting the microstructure and the columnar-to-equiaxed transition. 

## Figures and Tables

**Table 1:** Thermophysical properties and modeling parameters of NH_4_Cl-H_2_O [41].

Property/Parameters	Symbol	Unit	Value
Concentration at the equilibrium	$C_{eq}$ (T = 25 °C)		0.2771
Solid concentration	$C_{s}$		1
Liguid and solid densities	$\rho_{l};\;\rho_{S}$	$kg/m^{3}$	1090; 1520
Liquid diffusion coefficient	D	(m^2^ s^−1^)	3.2 × 10^−9^

**Table 2:** Mean percentage error by which predictions of the models (Equations (2) and (12)) differ from the experimental minima.

Models	Ivantsov (All Data Range)	Ivantsov (Ω < 0.0065)	McFadden and Corriel (Data with Ω < 0.0125) (β = 0.4; K = 0)	McFadden and Corriel (All Data Range) (β = 0.4; K = 0)	McFadden and Corriel (All Data Range) (β = 0.4; K = 100)
Mean percentage error	>100%	48%	14%	42%	2%

## Data Availability

Data is contained within the article.

## Nomenclature

$C_{0}$	Concentration	V	Growth rate (m/s)
C_s_	Solid concentration	K	Interfacial kinetic coefficient
C_eq_	Concentration at the equilibrium	$\rho_{l},\rho_{s}$	Liquid and solid density (kg/m^3^)
Ω	Supersaturation	β	Relative density ratio
Ω_K_	Kinetic supersaturation	Pe	Peclet number
D	Liquid diffusion coefficient (m^2^ s^−1^)	E_1_	Exponential integral function
R	The radius of the tip dendrite (m)	Γ	Complementary incomplete gamma function
